# Attitudes towards COVID-19-Related Medical Misinformation among Healthcare Workers and Non-Healthcare Workers in Saudi Arabia during the Pandemic: An Online Cross-Sectional Survey

**DOI:** 10.3390/ijerph18116123

**Published:** 2021-06-06

**Authors:** Amna Abdullah Alotiby, Laila Naif Al-Harbi

**Affiliations:** 1 Department of Haematology and Immunology, Faculty of Medicine, Umm Alqura University, Makkah 24237, Saudi Arabia; aamogaty@uqu.edu.sa; 2 Adipogenesis and Immunobiology Research Lab, Department of Food Science and Nutrition, College of Food Science and Agriculture, King Saud University, Riyadh 11451, Saudi Arabia

**Keywords:** COVID-19, Saudi Arabia, misinformation, behaviour, infodemic, infodemiology

## Abstract

Since the SARS-CoV-2 virus caused a global pandemic, the amount of misinformation in various media outlets has been on the rise. This has caused confusion among both healthcare workers and the general population about what the proper precautions against COVID-19 are. This study investigated attitudes towards misinformation related to protective measures that can be taken against COVID-19. The study was conducted in Saudi Arabia using an online survey questionnaire during the first three months of lockdown responding to the pandemic. The sample size of the study was N = 1294, of which 275 were healthcare workers and 974 were non-healthcare workers. The findings indicate that the Saudi Arabian population has a “Neutral” attitude towards COVID-19-related misinformation, meaning that, overall, they neither agree nor disagree with the most common COVID-19-related misinformation. Both healthcare workers and non-healthcare workers displayed a “Neutral” attitude towards herbal remedies for COVID-19. The level of agreement regarding the SARS-CoV-2 virus remaining in the throat for two days and the BCG vaccine protecting against COVID-19 was low. The findings of this research imply that knowledge dissemination is severely lacking in Saudi Arabia and that the Ministry of Health in Saudi Arabia should sincerely consider educating healthcare workers better about verified and true information regarding COVID-19. Conclusion: Future research should include larger sample sizes for each of the healthcare specialties surveyed in this study and analyse their attitudes towards COVID-19 misinformation.

## 1. Introduction

The SARS-CoV-2 virus can infect patients with the COVID-19, the severity of which can range from being completely void of symptoms to being fatal [1,2,3]. After COVID-19 was declared a global pandemic by the WHO in March 2020 [3], misinformation regarding causes, cures, and treatments of COVID-19 appeared in several media outlets. In an attempt to curb the spread of misinformation, the World Health Organisation (WHO) has created a webpage about the common misconceptions of COVID-19 [4]; however, several of the unsupported claims about protective measures against COVID-19 continue to be disseminated.

Misinformation surrounding COVID-19 has led to confusion, lack of consensus, and a failure to take the severity of the coronavirus pandemic as seriously as it should have been by the general public [5]. For instance, misinformation circulated heavily in social media [6] and, with not enough resources available to label all misinformation as false, or unverified, herbal remedies started providing a false sense of security to the public [7]. Instead of focusing on actual preventive measures such as social distancing, wearing face masks, and regularly washing hands with soap or sanitising with 70% isopropyl alcohol [8], focusing on alternative solutions not only hinders progress in medical science but also poses significant health risks. For instance, several cases of chloroquine poisoning were reported in Nigeria in March 2020, after the then President of the United States, Donald Trump, endorsed it as a treatment for COVID-19 [9]. 

One of the more commonly observed items of misinformation was based on the herbal properties of garlic and onions. Some studies support the idea that food with herbal properties can help improve the body’s immune system if taken over a long time, with regular consistency and supplemented by a good and healthy lifestyle [10,11,12,13]. As of yet, there is no evidence to support the hypothesis that herbal properties can cure the infections caused by SARS-CoV-2 or that the consumption of garlic and onions can immediately boost the immune system. In addition to misinformation about herbal medicines, two more items of misinformation were observed that related to COVID-19. The first was that coronavirus remains in the throat for a day or two and can be gargled away with hot, salty water [14], and the second was that the Bacillus Calmette–Guérin vaccine for tuberculosis or the seasonal flu shot can protect against COVID-19 infections [15,16]. It has been posited that attitudes toward medical misinformation and rumours could be independent of an individual’s level of education when it comes to the use of herbal medicine [13]. To the best of the authors’ knowledge, there are no previous studies that compare the attitudes of healthcare workers (HCWs) to those of non-healthcare workers (NHCWs) regarding medical misinformation and herbal medicine. Therefore, this study was conducted to compare the attitudes toward medical misinformation of both groups and identify if associations exist between sociodemographic characteristics, HCWs’ specialties and a belief in misinformation. HCWs’ attitudes towards medical misinformation shed light on how prevalent misinformation is in Saudi Arabia among medical professionals.

## 2. Materials and Methods

### 2.1. Study Design

In order to better understand to what extent people agree or disagree with the most common COVID-19-related misinformation, it is imperative that a cross-sectional study be conducted in a region where there is a high risk of misinformation being spread. This research was therefore conducted in Saudi Arabia, where herbal medicines are considered to be remedies against common diseases [10]. This research tested attitudes toward herbal medicines and two types of common misinformation about COVID-19.

A quantitative research methodology was used to design this cross-sectional study. This study took a positivist approach and made no assumptions about the expected associations between sociodemographic characteristics and attitudes towards medical misinformation. The sociodemographic constructs in this study consisted of region, nationality, gender, age group, and education level. The medical misinformation constructs consisted of the following items: garlic cures infections caused by COVID-19, onions cure infections caused by COVID-19, SARS-CoV-2 remains in the throat for two days and can be removed by gargling with salty water, and being vaccinated against TB also serves as protection against COVID-19. This study was conducted on both HCWs and NHCWs. There were no separate categorisations of NHCWS, but HCWs were further categorised to gain more information about the types of medical professions and their association with misinformation regarding COVID-19. The measurement tool used to conduct this study is discussed in the next section. 

### 2.2. Measurement Tool

An online survey was constructed for this study. The survey was broken down into two parts. The first part consisted of seven items and pertained to the sociodemographic characteristics of the survey participants. Item one established whether the survey respondent was an HCW or an NHCW. Item two enquired about the specialty of the HCW. Item three asked about the participants’ region of residence in Saudi Arabia, with five choices: central, eastern, northern, southern, and western. Item four asked about the nationality of the participant. Item five asked about gender, item six about age, and item seven about education level.

The second part of the questionnaire had five items pertaining to common misinformation regarding COVID-19. The second part of the questionnaire had a five-point Likert scale response choice for each of the five items. Items 1 and 2 asked the participants to rank their level of agreement with the use of garlic and onion, respectively, as protection against COVID-19. Item 3 asked for the participants’ level of agreement regarding the misinformation that SARS-CoV-2 remains in the throat for two days and can be gargled away with salty water. Item 4 asked about the participants’ level of agreement regarding the misinformation that the BCG vaccine can protect people from COVID-19. Item 5 asked how often the participants felt that watching videos on natural remedies was helpful during the COVID-19 pandemic.

The questionnaire was presented to the general population of Saudi Arabia and HCWs during the first three months of the lockdown responding to the pandemic, between April and June, via social media platforms and an email sent by the Saudi Commission for Health Specialties (SCFHS). The emails were sent specifically to HCWs who were registered with the SCFHS database.

This questionnaire was developed with an assistant professor in immunology and went through iterative improvements through various stages of verification with three experts in public health, immunology, and clinical nutrition. The questionnaire was designed in English and translated into Arabic, as this was the native language of the participants. The final Arabic questionnaire was pre-tested by the three experts (who were native speakers of Arabic) and 20 volunteers, a mix of NHCWs and HCWs. The final questionnaire was modified and further pre-tested by sending it to 100 random target samples to determine if respondents understood the questions, could perform the tasks, and had the information the questions required.

### 2.3. Study Population

The study population for this research were residents of Saudi Arabia. Thus, the study population size was 34 million [17] and the number of HCWs in Saudi Arabia is 500,000 [18]. However, since both HCWs and NHCWs were required, and in order to ensure that the data received had a confidence interval of 95% with a 5% margin of error, the sample size required for this study was N = 770 [19], of which N = 385 were HCWs and N = 385 were NHCWs. Participants were included in this study if they were aged 18 years and above, whereas they were excluded if they were aged below 18 years.

### 2.4. Data Analysis Tools

After the data were collected, they were pre-processed, then coded and saved as a file format compatible with IBM SPSS 22 (SPSS, Inc., Chicago, IL, USA). Age groups were categorised using SPSS as “Young adults” for the age range from 18 to 30, “Middle-aged adults” for the age range from 31 to 50, and “Older adults” for the age range from 51 to 80. The attitude section included five statements and the responses were assessed using a five-point Likert scale—“strongly disagree,” “disagree,” “neutral”, “agree”, and “strongly agree“—with each weighted from 1 to 5, respectively. The attitude score was calculated by summing up all the discrete scores for attitude items. The total score ranged from 5 to 25 points. The overall attitude was classified into three levels for negative, neutral and positive attitudes. Participants who had scores of 5–11 were considered to have a “Negative attitude”, those who had scores of 12–18 were considered to have a “Neutral attitude”, and those who had scores of 19–25 were considered to have a “Positive attitude” towards medical misinformation regarding COVID-19. A negative attitude meant that the participants disagreed with the information, while a positive attitude meant they agreed with the misinformation. A neutral attitude meant that the participants were undecided, neither agreeing nor disagreeing with the misinformation. Comparative analysis between study groups (HCWS and NHCWs) was done for all variables, including for sociodemographic data, and attitude items regarding medical misinformation pertaining to the protective measures against COVID-19 infection. The significance of relations in cross-tabulation was tested using a Pearson chi-square test or exact probability test for small frequencies.

## 3. Results

### 3.1. Participant Demographic Characteristics

The total number of participants for this study was N = 1249, of which 275 (22%) were HCWs and 974 (78%) were NHCWs. The majority of the survey participants resided in the Western and Central Regions of Saudi Arabia, comprising of 44.8% and 32.3% of the sample population, respectively. Most of the survey participants were Saudi Arabian nationals, comprising of 95.4% of the sample population. The survey population consisted of more females (76.9%) than males (23.1%). There were more responses from young adults (66.0%) than middle-aged (31.3%) or older (2.7%) adults. Around 57.1% of the survey population had a bachelor’s degree, 26.1% had a post-graduate degree, 14.3% had a high school education, and 2.6% had less than high school education.

The detailed demographic distribution of the survey participants is provided in Table 1, including details for HCWs and NHCWs.

### 3.2. Attitudes towards Medical Misinformation

The attitudes towards medical misinformation among HCWs and NHCWs are illustrated in Table S1 in the Supplementary Materials. The first column describes the nature of the misinformation. The five-point Likert scale response is represented beside each of the misinformation details. The scales are strongly disagree (SD), disagree, neutral, agree, and strongly agree (SA).

Around 51% of the survey participants responded that they “strongly agree” or “agree” with the misinformation that garlic and onions are better than other natural products to protect from COVID-19 infection. More than 41% of HCWs who responded to the survey also agreed with both the pieces of misinformation, while only 20.8% of surveyed HCWs indicated “disagree” or “strongly disagree”. More than 38% of HCWs did not have an opinion regarding the misinformation. Of the surveyed NHCWs, 53.8% replied that they “strongly agree” or “agree” with the misinformation, only 17.4% that they “strongly disagree” or “disagree”, and 28.9% were neutral about the misinformation. These results were statistically significant at *p* = 0.001.

Regarding misinformation about coronavirus staying in the throat for two days and being able to remove it by gargling with salt water, the majority of the surveyed population disagreed with this misinformation. More than 64% responded with “strongly disagree” or “disagree”, 15.9% said they were “neutral”, and only 19.8% replied with “agree” or “strongly agree”. Of the surveyed population, 74.6% of HCWs did not agree with the misinformation, 11.6% were neutral, and 13.8% agreed with it. In case of NHCWs, 61.4% did not agree with the misinformation, 17% were neutral about it, and 21.6% agreed. These results were statistically significant at *p* = 0.001.

The majority of the surveyed population did not agree with the misinformation that pre-existing vaccines work against the coronavirus that causes COVID-19. Overall, 74.3% of the surveyed population replied that they “strongly disagree” or “disagree” with this misinformation, 16.4% were neutral about it, and only 9.3% said that they “strongly agree” or “disagree” with the misinformation. Among the surveyed HCWs, 76.4% said that they “strongly disagree” with the misinformation, 12% were neutral about it, and only 8.7% agreed with the misinformation. Among the NHCWs, 67.7% indicated that they “strongly disagree”, 17.7% were “neutral” and only 9.4% replied with “disagree” or “strongly disagree” with regard to the misinformation. However, these values were not statistically significant at *p* = 0.058.

The majority of the surveyed population, 42.4%, believed that it was “sometimes” useful to watch videos talking about using natural remedies as preventive medicine during the COVID-19 pandemic. More than 44% of HCWs and 41% of NHCWs considered that watching these videos was “sometimes” useful, while 21.5% of HCWs and 13.6% of NHCWs believed that watching these videos was “never” helpful. These results were statistically significant at *p* = 0.002.

Aggregating the results together, it can be seen that, overall, 32.5% of the surveyed population scored their levels of agreement for the listed misinformation between 5–11, meaning they had a negative attitude towards misinformation; 56.4% scored 12–18, meaning they had a neutral attitude towards misinformation; and 11.0% scored 19–25, meaning they had a positive attitude towards misinformation. Among the HCWs, 44.7% had a negative attitude, 48.0% had a neutral attitude, and 7.3% had a positive attitude towards misinformation. Among the NHCWs, 29.1% had a negative attitude, 58.8% had a neutral attitude, and 12.1% had a positive attitude towards misinformation. The overall attitudes and their association with sociodemographic characteristics are explained in the next section.

### 3.3. Association between Medical Misinformation and Socio-Demographic Characteristics

Key observations on the association between attitudes towards medical misinformation and participant sociodemographic characteristics are presented in this section. Overall, it was observed that, for any region, nationality, gender, age group, or education level, the majority of the surveyed population had a neutral attitude towards COVID-19-related misinformation, and there were more people who held a negative attitude towards medical misinformation than those who held a positive attitude. However, only the data for nationality (*p* = 0.027) and age group (*p* = 0.001) were statistically significant. Table 2 provides details of the frequency and percentages of the sociodemographic distributions of the participants according to their attitudes towards COVID-19-related medical misinformation. The first column lists the sociodemographic indicator. The categorisation of these sociodemographic indicators is given on the adjacent column to their right, followed by the distribution according to attitudes towards medical misinformation.

The final row, healthcare specialty, was statistically significant at *p* = 0.001. This row shows the attitudes towards medical misinformation among different HCWs. Physicians and laboratory technologists were the most common healthcare specialties, followed by clinical nutritionists and pharmacists. The majority of surveyed physicians, 51.5%, had a neutral attitude, 44.4% had a negative attitude, and only 4% had a positive attitude towards medical misinformation. The majority of laboratory technologists, 53.8%, had a negative attitude towards medical misinformation, 43.6% were neutral, and only 2.6% had a positive attitude towards medical misinformation. In total, there were 275 HCWs, of which 123 (44.7%) displayed negative attitudes towards medical misinformation, 132 (48%) displayed neutral attitudes towards misinformation, and only 20 (7.3%) displayed positive attitudes towards medical misinformation. The discussion pertaining to these results is elaborated in the next section.

## 4. Discussions

It is a cause for concern that the Saudi Arabian population does not have a stronger level of disagreement regarding COVID-19-related misinformation. For instance, overall, both HCWs and NHCWs had a “Neutral” attitude towards the most commonly circulated medical misinformation about fighting COVID-19 infection, meaning that they neither agreed nor disagreed with the misinformation regarding COVID-19. Ideally, the level of the “Negative” attitude should be much higher than both the “Neutral” and “Positive” attitudes, especially among HCWs. The concerning findings of this study are consistent with similar studies regarding misinformation in Saudi Arabia during the COVID-19 pandemic.

The first concern is that, even though hygiene practices of the Saudi Arabian general population have improved overall and there is evidence of increased social distancing and hygiene practices [20], attitudes towards misinformation have not improved. This study agrees with other studies in the literature that suggest that myths and misinformation among the Saudi Arabian general population are prevalent [21] and that the majority of Saudi Arabians are prone to believing the misinformation they see across various media channels [22]. Comparing the state of misinformation spread in Saudi Arabia to that in the rest of the world also present further points of discussion.

In Italy, there was a heavy spread of misinformation during the early stages of the pandemic among the general population [23], and in South Korea the spread of and belief in misinformation during the early stages of the COVID-19 pandemic was observed to be as high as 41% [24]. A global study conducted among parents indicated that stress and lack of definitive answers, especially among parents whose children had fatal illnesses, were seen to be very high [25]. This made them vulnerable and susceptible to medical misinformation, as their uncertainty prompted them to explore all possibilities, including those which might be misinformative. Therefore, other countries also have the problem of their populations believing in medical misinformation [26].

A big concern for Saudi Arabia is that, one year into the pandemic, the general population’s misplaced trust in herbal medicine is still evidently observable. A bigger concern is that HCWs in Saudi Arabia still believe in medical misinformation, albeit a small portion. One of the reasons why this small portion of HCWs still believes in medical misinformation could be due to the findings of some preliminary studies, which showed some plausibility for unconfirmed remedies. Explicitly placing faith in the preliminary studies, combined desperation in the search for remedies, can lead HCWs to believe in the misinformation. Thus, the faith in herbal remedies among Saudi Arabian HCWs could be based on the preliminary supportive data regarding improved immune systems from garlic and onion consumption. This is still a cause of concern because, while consuming herbs should not be discouraged, suggesting herbs as a remedy should not be the primary response to COVID-19. Comparing the responses of HCWs and NHCWs also presents further causes of concern.

The level of agreement among HCWs and NHCWs regarding herbal remedies for protection against COVID-19 infection was high. This suggests that the same misinformation that the general public are inclined to believe in is also equally likely to affect HCWs. Being well-informed about COVID-19 and its causes, effects, and remedies is pertinent to the jobs of HCWs, especially when worldwide COVID-19 cases continue to rise daily. The observation that HCWs and NHCWs both agree with the same misinformation suggests a severe gap in knowledge and awareness among HCWs in Saudi Arabia. However, the belief in herbal remedies could be accounted for by cultural beliefs in Saudi Arabia regarding herbal medicinal practices [10]. Thus, while misinformation regarding herbal remedies runs high in the Saudi Arabian population, people do not agree about more crucial misinformation, such as SARS-CoV-2 staying in the throat for two days, or that being vaccinated with the BCG vaccine can work to protect people from COVID-19. Both HCWs and NHCWs did not agree with this misinformation.

Focusing on the medical specialties of the HCWs, the “Neutral” attitude to misinformation was indicative of the vulnerability of HCWs to believing medical misinformation. It is expected that HCWs should be well-informed about what is actual medical information and they should be able to advise patients based on supporting evidence and take a firm stance against medical misinformation. The availability of information made accessible by the WHO is a direct effort to curb the spread of misinformation [27]. However, the response of clinical nutritionists, laboratory technologists, pharmacists, and physicians, who neither strongly disagreed nor agreed with regard to COVID-19-related misinformation, reflects uncertainty and vulnerability. Public health promoters especially should not be displaying such a high level of agreement with COVID-19-related misinformation. Information and knowledge disseminated through public health promoters should be grounded on confirmed research findings. If the general public’s practices are based on medical misinformation, this can further the magnitude of the COVID-19 epidemic. Thus, public health promoters need to be better informed about COVID-19 as the nation’s safety depends on their work.

The sociodemographic characteristics indicated that both Saudi Arabians and non-Saudis, in all the five regions in Saudi Arabia, need stronger knowledge dissemination methods so that their level of agreement to COVID-19-related misinformation leans more strongly towards disagreement. Resources for knowledge dissemination should be focused on all age groups, with special attention given to older adults. Provision of verified information about COVID-19 should also be focused on people of all educational backgrounds. The results do not show that those with higher levels of education necessarily have lower levels of disagreement.

## 5. Conclusions

The overall findings indicate that while the Saudi Arabians, as a nation, do not wholly believe in the misinformation surrounding COVID-19, the “Neutral” stance that the majority of the nation display is concerning. While the coronavirus pandemic may eventually pass, the lessons learned from this pandemic are critical for future generations. Thus, by focusing on the sociodemographic characteristics, the Ministry of Health should pay special attention to reallocating resources towards debunking misinformation and promoting verified facts surrounding COVID-19. Additionally, academics should focus future research on the causes behind such low levels of disagreement with COVID-19 medical misinformation among the Saudi Arabian population. The findings of this study also highlight the importance of a quick and massive restructuring of the communication methods and attitude requirements for being an HCW in Saudi Arabia. The previous section suggested the possibility that the cultural significance of herbal remedies might promote agreement with medical misinformation. Future studies are recommended to investigate the association between cultural influences and the attitude of HCWs toward medical misinformation. The attitudes of HCWs toward medical misinformation should be improved to face challenges in the future. Therefore, the current study suggests that an educational program needs to be established to raise HCWs’ levels of awareness about medical misinformation and prepare them to deal wisely with this misinformation. The main limitation of the current study was the sample size of HCWs. Further studies might be useful if they acquire a large sample size for each of the healthcare specialties and include a larger questionnaire investigating more types of misinformation to obtain a better overview of HCWs’ responses. 

## Figures and Tables

**Table 1:** Distribution of study groups according to their personal characteristics. N = 1249; HCWs, healthcare workers; NHCWs, non-healthcare workers.

Personal Data	Total (%)	HCWs (*n* = 275)		NHCWs (*n* = 974)		*p*-Value *
		No.	%	No.	%	
**Region**						0.056
Central	403 (32.3%)	76	27.6%	327	33.6%	
Eastern	123 (9.8%)	27	9.8%	96	9.9%	
Northern	83 (6.6%)	8	2.9%	75	7.7%	
Southern	80 (6.4%)	23	8.4%	57	5.9%	
Western	560 (44.8%)	141	51.3%	419	43.0%	
**Nationality**						0.114
Saudi	1192 (95.4%)	254	92.4%	938	96.3%	
Non-Saudi	57 (4.6%)	21	7.6%	36	3.7%	
**Gender**						0.001
Male	288 (23.1%)	98	35.6%	190	19.5%	
Female	961 (76.9%)	177	64.4%	784	80.5%	
**Age group**						0.156
Young adults (18–30)	824 (66.0%)	193	70.2%	631	64.8%	
Middle-aged adults (31–50)	391 (31.3%)	70	25.5%	321	33.0%	
Older adults (51–80)	34 (2.7%)	12	4.4%	22	2.3%	
**Education level**						0.001
Less than high school	32 (2.6%)	0	0.0%	32	3.3%	
High school	178 (14.3%)	3	1.1%	175	18.0%	
Bachelor’s degree	713 (57.1%)	142	51.6%	571	58.6%	
Post-graduate degree	326 (26.1%)	130	47.3%	196	20.1%	

* Pearson chi-square test or exact probability test as appropriate. Differences were considered significant at *p*-value < 0.05.

**Table 2:** Distribution of participants’ attitudes towards medical misinformation regarding the protective measures against COVID-19 infection in relation to their personal data.

Demographic Data		Attitude Level						*p*-Value *
		Negative		Neutral		Positive		
		No.	%	No	%	No.	%	
**Region**	Central	152	37.7%	219	54.3%	32	7.9%	0.075
	Eastern	43	35.0%	65	52.8%	15	12.2%	
	Northern	20	24.1%	52	62.7%	11	13.3%	
	Southern	21	26.3%	47	58.8%	12	15.0%	
	Western	170	30.4%	322	57.5%	68	12.1%	
**Nationality**	Non-Saudi	11	19.3%	42	73.7%	4	7.0%	0.027
	Saudi	395	33.1%	663	55.6%	134	11.2%	
**Gender**	Male	84	29.2%	170	59.0%	34	11.8%	0.383
	Female	322	33.5%	535	55.7%	104	10.8%	
**Age group**	Young adults (18–30)	309	37.5%	440	53.4%	75	9.1%	0.001
	Middle-aged adults (31–50)	91	23.3%	244	62.4%	56	14.3%	
	Older adults (51–80)	6	17.6%	21	61.8%	7	20.6%	
**Educational level**	Less than high school	8	25.0%	20	62.5%	4	12.5%	0.155
	High school	44	24.7%	117	65.7%	17	9.6%	
	Bachelor’s degree	238	33.4%	396	55.5%	79	11.1%	
	Post-graduate degree	116	35.6%	172	52.8%	38	11.7%	
**Healthcare specialty**	Clinical nutrition	11	68.8%	4	25.0%	1	6.3%	0.001
	Health Administrator	2	13.3%	10	66.7%	3	20.0%	
	Laboratory technologist	42	53.8%	34	43.6%	2	2.6%	
	Medical care and rehabilitation	1	16.7%	5	83.3%	0	0.0%	
	Nurse	7	33.3%	9	42.9%	5	23.8%	
	Paramedical	3	50.0%	3	50.0%	0	0.0%	
	Pharmacist	10	43.5%	12	52.2%	1	4.3%	
	Physician	44	44.4%	51	51.5%	4	4.0%	
	Public health promotion	1	16.7%	3	50.0%	2	33.3%	
	Radiologist	2	40.0%	1	20.0%	2	40.0%	

* Pearson chi-square test or exact probability test as appropriate. Differences were considered significant at *p*-value < 0.05.

## Data Availability

The original contributions presented in the study are included in the article; further inquiries can be directed to the corresponding author. Further information and requests for resources should be directed to and will be fulfilled by the Lead Contact, Alotiby A (aamogaty@uqu.edu.sa).

## Supplementary Materials

The following are available online at https://www.mdpi.com/article/10.3390/ijerph18116123/s1, Table S1: Distribution of study groups attitude towards medical misinformation regarding the protective measures against the COVID-19 infection. N = 1249, SD = Strongly Disagree, SA = Strongly Agree, (HCWs) healthcare workers, (NHCWs) non-healthcare workers
