# Supplementary material for: Attitudes towards COVID-19-Related Medical Misinformation among Healthcare Workers and Non-Healthcare Workers in Saudi Arabia during the Pandemic: An Online Cross-Sectional Survey
Source: Int J Environ Res Public Health. 2021 Jun 6;18(11):6123. doi: 10.3390/ijerph18116123 (PMC8201028; doi:10.3390/ijerph18116123)
Supplement: Supplementary file 1 [file ijerph-18-06123-s001.zip › ijerph-1224696-supplementary.pdf]

Attitude towards Covid-19 Related Medical Misinformation Among Health care workers and Non-Health care workers in Saudi Arabia during the pandemic: An Online Cross-Sectional Survey

Amna Abdullah Alotiby[1] and Laila Naif Al-Harbi[2*]

1 Department of Haematology and Immunology; Faculty of Medicine, Umm Alqura University; Makkah; Saudi Arabia
2 Adipogenesis and Immunobiology Research Lab, Department of Food Science and Nutrition; College of Food Science and Agriculture, King Saud University; Riyadh, 11451; Saudi Arabia.

Supplementary material

Table S1. Distribution of study groups attitude towards medical misinformation regarding the protective measures against the COVID-19 infection. N= 1249, SD= Strongly Disagree, SA= Strongly Agree, (HCWs) healthcare workers ,(NHCWs) non-healthcare workers.

		Total		HCWs		NHCWs		P-value*
		No.	%	No.	%	No.	%	
Garlic works more than other natural products to protect from COVID-19 infection	SD	42	3.4%	15	5.5%	27	2.8%	.001
	Disagree	184	14.7%	42	15.3%	142	14.6%	
	Neutral	386	30.9%	105	38.2%	281	28.9%	
	Agree	494	39.6%	86	31.3%	408	41.9%	
	SA	143	11.4%	27	9.8%	116	11.9%	
Onion works more than other natural products to protect from the COVID-19 infection	SD	42	3.4%	15	5.5%	27	2.8%	.001
	Disagree	184	14.7%	42	15.3%	142	14.6%	
	Neutral	386	30.9%	105	38.2%	281	28.9%	
	Agree	494	39.6%	86	31.3%	408	41.9%	
	SA	143	11.4%	27	9.8%	116	11.9%	
Coronavirus remains in the throat for a day or two, and if a person has gargled with water and salt, it eliminates it before it reaches the lungs and prevents contracting the infection	SD	715	57.2%	190	69.1%	525	53.9%	.001
	Disagree	88	7.0%	15	5.5%	73	7.5%	
	Neutral	198	15.9%	32	11.6%	166	17.0%	
	Agree	94	7.5%	17	6.2%	77	7.9%	
	SA	154	12.3%	21	7.6%	133	13.7%	
The use of other pre-existing vaccines can protect people from infection from Coronavirus, such as tuberculosis vaccine or seasonal influenza vaccine	SD	869	69.6%	210	76.4%	659	67.7%	.058
	Disagree	59	4.7%	8	2.9%	51	5.2%	
	Neutral	205	16.4%	33	12.0%	172	17.7%	
	Agree	55	4.4%	10	3.6%	45	4.6%	
	SA	61	4.9%	14	5.1%	47	4.8%	
Videos talking about using natural remedies as preventive medicine during the COVIOD-19 pandemic are useful	Never	191	15.3%	59	21.5%	132	13.6%	.002
	Sometimes	529	42.4%	123	44.7%	406	41.7%	
	Often	133	10.6%	28	10.2%	105	10.8%	
	Usually	182	14.6%	32	11.6%	150	15.4%	
	Always	214	17.1%	33	12.0%	181	18.6%	
Overall attitude	Negative	406	32.5%	123	44.7%	283	29.1%	.001
	Neutral	705	56.4%	132	48.0%	573	58.8%	
	Positive	138	11.0%	20	7.3%	118	12.1%	

* Pearson chi-square test or exact probability test as appropriate,
The difference is significant at P value < 0.05.
